# ﻿Four new species of *Tapinocyba* Simon, 1884 (Araneae, Linyphiidae) from Jiangjin District of Chongqing, China

**DOI:** 10.3897/zookeys.1219.133899

**Published:** 2024-12-03

**Authors:** Muhammad Irfan, Dai Yun, Wang Lu-Yu, Zhang Zhi-Sheng

**Affiliations:** 1 Key Laboratory of Eco-environments in Three Gorges Reservoir Region (Ministry of Education), School of Life Sciences, Southwest University, Chongqing 400715, China; 2 College of Plant Protection, Southwest University, Chongqing 400715, China; 3 Forestry Bureau of Jiangjin District, Chongqing 402262, China

**Keywords:** Description, Erigoninae, morphology, sheet-web, taxonomy

## Abstract

Four new species of the genus *Tapinocyba* Simon, 1884 are described from Jiangjin, Chongqing: *T.centralis***sp. nov.** (♂), *T.denticulata***sp. nov.** (♂♀), *T.triangularis***sp. nov.** (♂♀), and *T.virga***sp. nov.** (♂♀). The new species exhibit distinctive genital features, such as a bifurcated embolus tip in the male palp of *T.denticulata***sp. nov.** and *T.virga***sp. nov.**, and embolus tip unbifurcated in *T.centralis***sp. nov.** The epigynes display more unique characteristics, such as epigynal plate with a pit ventrally on its frontal face in *T.triangularis***sp. nov.** and *T.virga***sp. nov.**, which is absent in all other known *Tapinocyba* species. Detailed descriptions, along with photographs of genital characters, somatic features, and a distribution map, are provided.

## ﻿Introduction

The family Linyphiidae is one of the most diverse spider families worldwide, comprising 634 extant genera and 4,858 species, including 11 fossil genera and 62 species (WSC 2024). Currently, 532 species in 175 genera have been reported from China (Tanasevitch 2024), of which 37 species in 22 genera have been recorded in Chongqing Municipality (Irfan et al. 2022, 2023a, 2023b). *Tapinocyba* Simon, 1884, is a small genus consisting of 41 species, primarily distributed across Nearctic and Palearctic regions (WSC 2024). In the Chinese fauna, three species have been recorded, specifically from the provinces of Jilin, Sichuan, and Taiwan (Sha et al. 1994; Tanasevitch 2011, 2018).

Jiangjin District is located in the southwest of Chongqing, China, along the upper section of the Yangtze River. This work is the first study on Linyphiidae spiders collected in Jiangjin. As a result, four new species belonging to the genus *Tapinocyba* have been identified and described here.

## ﻿Materials and methods

The specimens were collected using Malaise traps and sieving leaf litter methods. All specimens were preserved in 75% ethanol. Left male palps were examined and photographed after dissection. After dissection, epigynes were cleared in trypsin enzyme solution before examination and photography. The specimens were examined and measured using Leica M205A stereomicroscope equipped with Leica DFC450 camera and LAS v. 4.6 software. All the photos of habitus and genitalia were taken with Kuy Nice CCD mounted on an Olympus BX53 compound microscope. Compound focus images were generated using Helicon Focus v. 6.7.1. Eye sizes were measured at the maximum dorsal diameter. Leg measurements are shown as total length (femur, patella, tibia, metatarsus, tarsus). All measurements are given in millimeters. Map was created using the online mapping software SimpleMappr (Shorthouse 2010) (Fig. 12). Specimens are deposited in the School of Life Sciences, Southwest University, Chongqing (**SWUC**), China. The terminology used in figures legend follows Hormiga (2000). In text “Fig.” and “Figs” refer to figures herein, while “fig.” and “figs” refer to figures published elsewhere.

The following abbreviations are used in the text and figures: **a.s.l.** = above sea level; **AER** = anterior eye row; **ALE** = anterior lateral eyes; **AME** = anterior median eyes; **AME–ALE** = the distance between AME and ALE; **AME–AME** = the distance between AMEs; **ARP** = anterior radical process; **CD** = copulatory ducts; **CO** = copulatory openings; **DP** = dorsal plate; **DSA** = distal suprategular apophysis; **DTA** = dorsal tibial apophysis; **E** = embolus; **FD** = fertilization ducts; **MM** = median membrane sensu van Helsdingen (1965) = embolic membrane sensu van Helsdingen (1986) and Hormiga (1994); **MSA** = marginal suprategular apophysis; **PC** = paracymbium; **PER** = posterior eye row; **PLE** = posterior lateral eyes; **PME** = posterior median eyes; **PME–PLE** = distance between PME and PLE; **PME–PME** = distance between PMEs; **PT** = protegulum; **R** = radix; **S** = spermatheca; **SPT** = suprategulum; **ST** = subtegulum; **T** = tegulum; **TmI** = position of trichobothrium on metatarsus I; **TP** = tailpiece; **VP** = ventral plate.

## ﻿Taxonomy

### ﻿Family Linyphiidae Blackwall, 1859

**Subfamily Erigoninae Emerton, 1882**

#### ﻿Genus Tapinocyba

Taxon classificationAnimaliaAraneaeLinyphiidae

Simon, 1884

86CFD030-D2F9-52AF-89E1-6CB70B3B87A1

##### Type species.

*Walckenaerapraecox* O. Pickard-Cambridge, 1873; gender feminine.

#### ﻿ Tapinocyba centralis sp. nov.

Taxon classificationAnimaliaAraneaeLinyphiidae

7CF42E0A-5805-5EB6-A14A-644EC7A6DC3C

https://zoobank.org/101F9F9F-3E08-47D8-A050-6C5A6ABFC7BA

**Figure 1.**
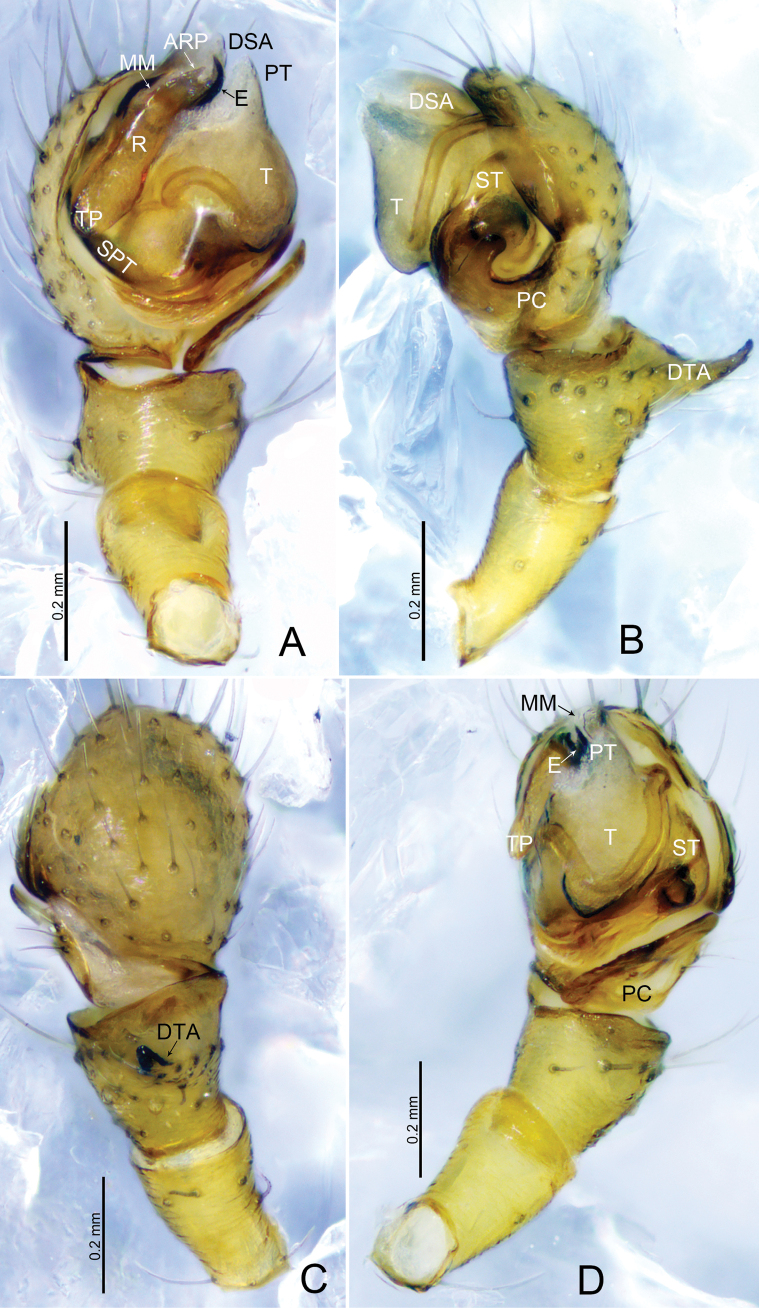
*Tapinocybacentralis* sp. nov., male holotype **A** palp, prolateral view **B** palp, retrolateral view **C** palp, dorsal view **D** palp, ventral view. Abbreviations: ARP = anterior radical process; DSA = distal suprategular apophysis; DTA = dorsal tibial apophysis; E = embolus; MM = median membrane; MSA = marginal suprategular apophysis; PC = paracymbium; PT = protegulum; R = radix; SPT = suprategulum; ST = subtegulum; T = tegulum; TP = tailpiece.

**Figure 2.**
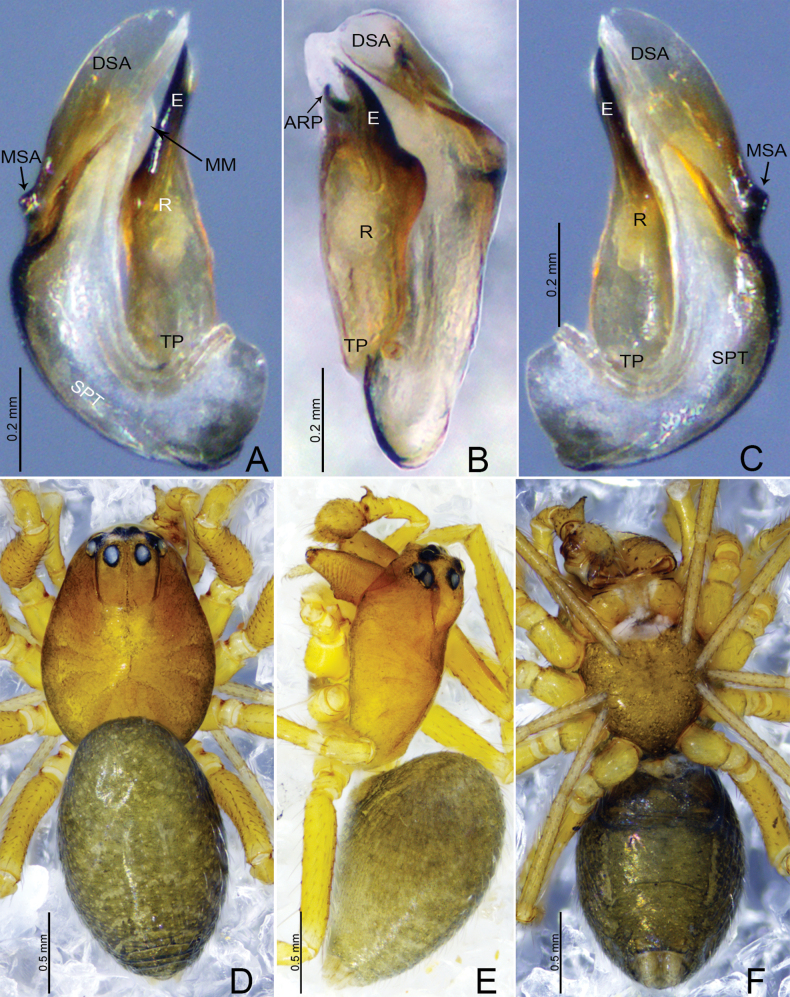
*Tapinocybacentralis* sp. nov., male paratype (**A–C**) and male holotype (**D–F**). **A–C** Embolic division **D** habitus, dorsal view **E** habitus, lateral view **F** habitus, ventral view. Abbreviations: ARP = anterior radical process; DSA = distal suprategular apophysis; E = embolus; MM = median membrane; MSA = marginal suprategular apophysis; R = radix; SPT = suprategulum; TP = tailpiece.

Figs 1 , 2 , 12 (中盾大蛛)

##### Type material.

***Holotype***: China • ♂; Chongqing Municipality, Jiangjin District, Zhuyang Town, Yunwuping, Guanyin Valley; 29°9′1.38″N, 105°57′28.18″E, 558 m a.s.l., 2023.I.7, Wang L.Y. et al. leg.; SWUC-T-LIN-26-01. ***Paratypes***: 3♂; same location data as holotype; SWUC-T-LIN-26-02 to SWUC-T-LIN-26-04 • 2♂; same location data as holotype; SWUC-T-LIN-26-02 & SWUC-T-LIN-26-03 • 1♂; Zhuyang Town, Yunwuping, Dashibao; 29°9′6.99″N, 105°57′34.51″E; 542 m a.s.l.; 2023.I.7; Wang L.Y. et al. leg.; SWUC-T-LIN-26-04.

##### Etymology.

The specific epithet is derived from the Latin adjective *centralis*, meaning “median” and referring to the dorsal tibial apophysis, which is located dorsally in almost median of the tibia in dorsal view of the male palp.

##### Diagnosis.

The male of *Tapinocybacentralis* sp. nov. resembles *T.praecox* (O. Pickard-Cambridge, 1873) in having the similar cephalic lobe and embolic division (Figs 1A–D, 2A–C; Hormiga 2000: fig. 27A–E, pl. 62A–F) and can be distinguished by the dorsal tibial apophysis located almost in median of tibia in dorsal view in *T.centralis* sp. nov. (Fig. 1C; vs located at the tip of tibia); anterior radical process present in *T.centralis* sp. nov. (Fig. 1C; vs absent).

##### Description.

**Male** (holotype, Fig. 2D–F): total length: 1.40. Carapace 0.76 long, 0.53 wide, brown, cephalic region strongly elevated, cephalic pits present behind the PLEs, fovea, cervical and radial grooves distinct. Clypeus 0.20 high. Chelicerae with five promarginal and five retromarginal teeth. Sternum yellowish brown as long as wide, shield-like, with microsetae. Eyes: AER recurved, PER procurved, slightly wider than AER, PMEs present on cephalic lobe. Eye sizes and interdistances: AME 0.03, ALE 0.06, PME 0.05, PLE 0.05, AME–AME 0.01, PME–PME 0.05, AME–ALE, 0.02, PME–PLE 0.06, AME–PME 0.08, ALE–ALE 0.24, PLE–PLE 0.25, ALE–PLE 0.01. Length of legs: I 1.93 (0.55, 0.20, 0.48, 0.38, 0.32), II 1.73 (0.47, 0.19, 0.40, 0.36, 0.31), III 1.46 (0.43, 0.17, 0.29, 0.32, 0.25), IV 1.97 (0.54, 0.18, 0.50, 0.41, 0.34). TmI 0.41 and TmIV absent. Tibial spine formula: 1-1-1-1. Opisthosoma 0.81 long, 0.52 wide, oval, greenish.

***Palp*** (holotype, Fig. 1A–D; a paratype, Fig. 2A–C). Femur unmodified, almost as long as both patella and tibia. Patella longer than tibia. Tibia widest at distal end, with one retrolateral and one dorsal trichobothria, dorsal tibial apophysis half the length of tibia, tapering towards tip with blunt end; paracymbium U-shaped, basally with setae, distal arm tip with blunt end; tegulum almost round, ventrally bulging above subtegulum, protegulum distinct, membranous; suprategulum small, with small median suprategular apophysis, invisible on unexpanded palp, distal suprategular apophysis membranous; embolic division simple, consisting of a radix longer than wide; tailpiece round; median membrane short; anterior radical process somewhat triangular with pointed end; embolus short, horn-shaped, slightly curved with pointed end.

**Female.** Unknown.

##### Distribution.

Known only from the type locality (Fig. 12).

#### ﻿ Tapinocyba denticulata sp. nov.

Taxon classificationAnimaliaAraneaeLinyphiidae

E31CE1AD-2378-5A71-A288-4C96933ED134

https://zoobank.org/F4C75A35-DB03-4B3F-9F54-4F640463163F

Figs 3 , 4 , 5 , 12

##### Type material.

***Holotype***: China • ♂; Chongqing Municipality, Jiangjin District, Simian Mountain, Zhengtian Valley, 28°36′46.97″N, 106°25′54.77″E, 1170 m a.s.l., 2023.III.02, Wang L.Y. et al. leg.; SWUC-T-LIN-27-01. ***Paratypes***: 1♀; same location data as holotype; SWUC-T-LIN-27-02 • 1♀: Simian Mountain, Chaoyuanguan, 28°38′53.38″N, 106°20′23.84″E, 920 m a.s.l., 2023.I.22, Wang B. et al. leg.; SWUC-T-LIN-27-03 • 1♂; Simian Mountain, Qinjiagou, 28°37′6.32″N, 106°23′53.40″E, 1131 m a.s.l., 2023.III.2, Zhang Z.G. et al. leg.; SWUC-T-LIN-27-04.

##### Etymology.

The specific epithet is derived from the Latin adjective *denticulatus*, meaning “teeth” and referring to the small teeth at the tip of dorsal tibial apophysis of the male palp.

##### Diagnosis.

**Figure 3.**
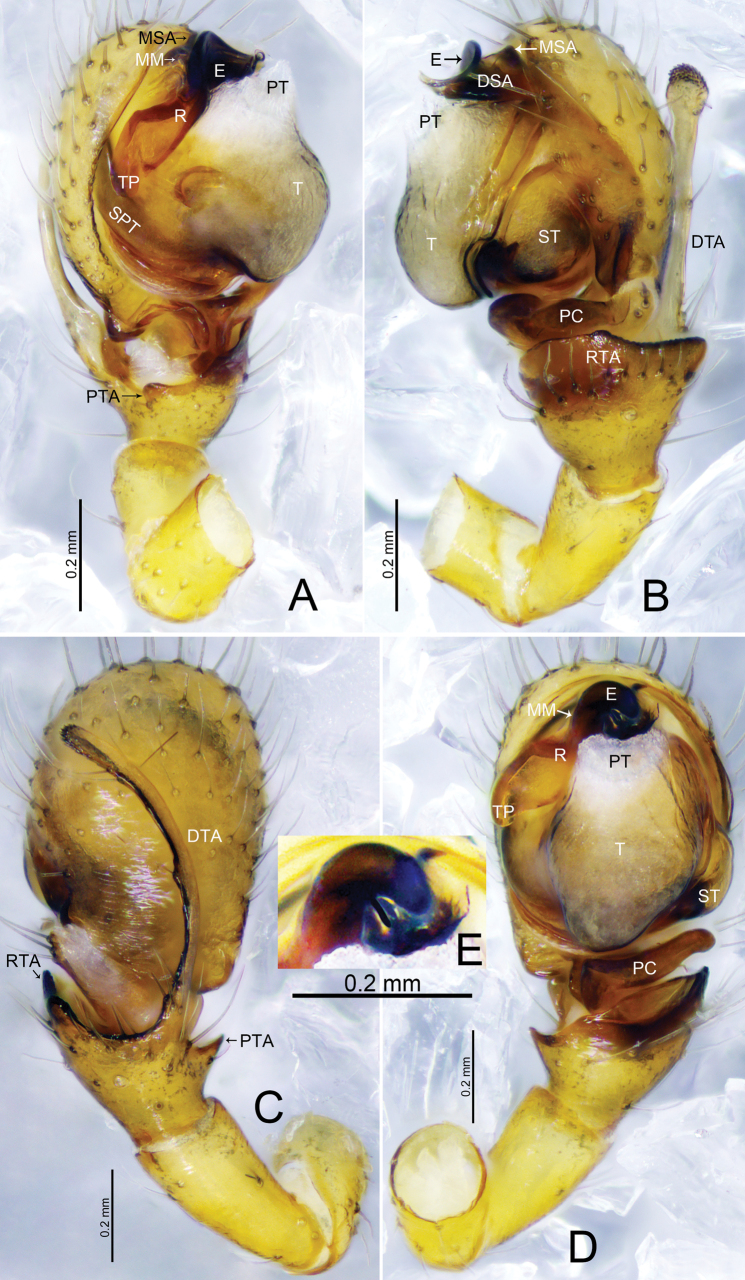
*Tapinocybadenticulata* sp. nov., male holotype **A** palp, prolateral view **B** palp, retrolateral view **C** palp, dorsal view **D** palp, ventral view **E** embolus, ventral view. Abbreviations: DSA = distal suprategular apophysis; DTA = dorsal tibial apophysis; E = embolus; MM = median membrane; MSA = marginal suprategular apophysis; PC = paracymbium; PT = protegulum; PTA = prolateral tibial apophysis; R = radix; RTA = retrolateral tibial apophysis; SPT = suprategulum; ST = subtegulum; T = tegulum; TP = tailpiece.

**Figure 4.**
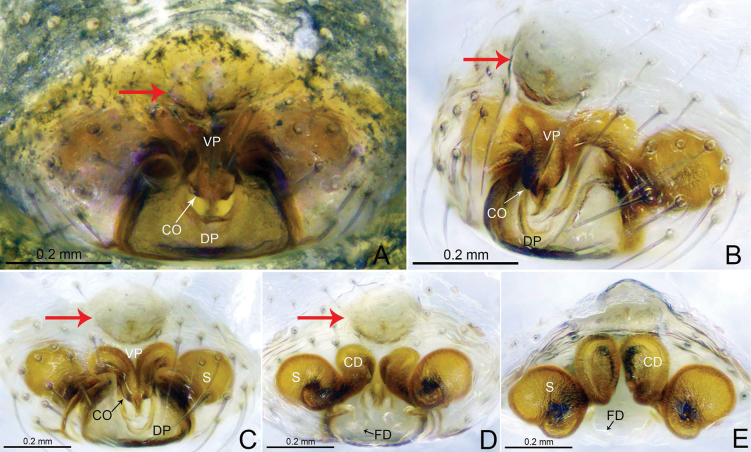
*Tapinocybadenticulata* sp. nov., female paratype **A, C** epigyne, ventral view **B** epigyne, lateral view **D** vulva, dorsal view **E** vulva, anterior view **A–D** red arrow indicating epigynal plate mid ventrally with a conspicuous round patch anteriorly. Abbreviations: CD = copulatory duct; CO = copulatory opening; DP = dorsal plate; FD = fertilization duct; S = spermathecae; VP = ventral plate.

**Figure 5.**
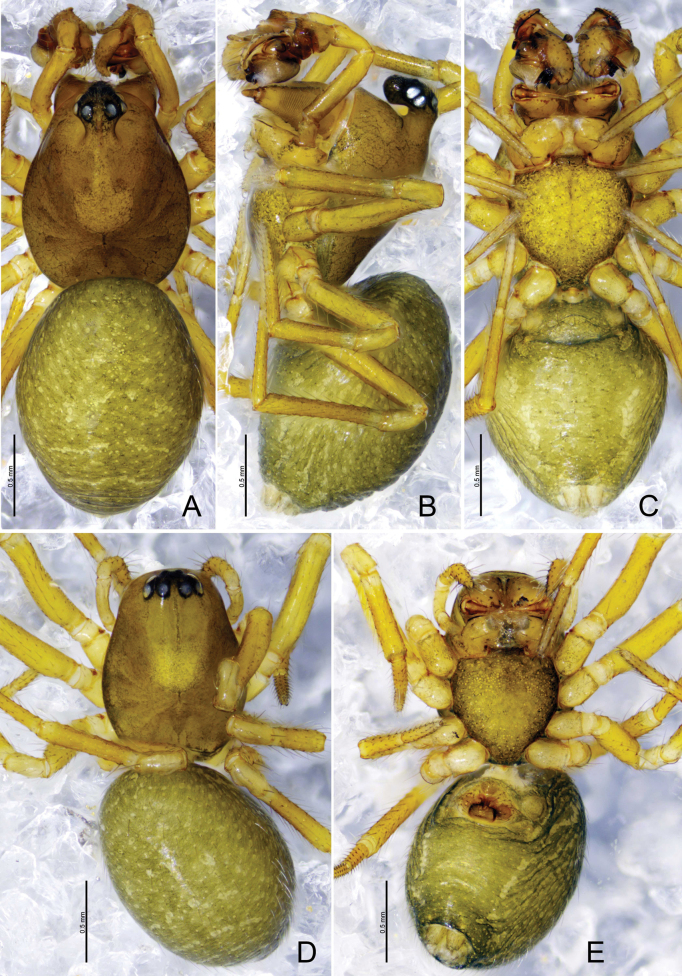
*Tapinocybadenticulata* sp. nov., male holotype (**A–C**) and female paratype (**D, E**) **A**, **D** habitus, dorsal view **B** habitus, lateral view **C, E** habitus, ventral view.

The male of *Tapinocybadenticulata* sp. nov. resembles *T.praecox* (O. Pickard-Cambridge, 1873) in having the similar embolic division (Fig. 3A–D; Hormiga 2000: fig. 27A–E, pl. 62A–F) and can be distinguished by the ocular region strongly curved, making right-angle with cephalothorax, extending above the clypeus in *T.denticulata* sp. nov. (Fig. 5B; vs ocular region not modified); dorsal tibial apophysis as long as cymbium, tip with small teeth, extending above the cymbium in *T.denticulata* sp. nov. (Fig. 3C; vs half the length of tibia, with blunt tip, extending away from cymbium); retrolateral tibial apophysis present in *T.denticulata* sp. nov. (Fig. 3C; vs absent); embolus curved clockwise with bifurcated tip in *T.denticulata* sp. nov. (Fig. 3A, B, E; vs slightly curved with blunt tip). The female of *T.denticulata* sp. nov. resembles *T.affinis* Lessert, 1907 in having the similar copulatory ducts and spermathecae (Fig. 4A–E; Wiehle 1960: figs 984a, b, 985) and can be distinguished by the tapering part of ventral plate as long as wide in *T.denticulata* sp. nov. (Fig. 4A, B, E; vs somewhat triangular). The epigyne of *Tapinocybadenticulata* sp. nov. can be distinguished from *T.praecox* by the dorsal plate ventrally grooved in *T.denticulata* sp. nov. (Fig. 4A–C; vs not grooved).

##### Description.

**Male** (holotype, Fig. 5A–C): total length 1.74; carapace 0.87 long, 0.60 wide, brown, cephalic region slightly elevated, cephalic pits absent, fovea, cervical and radial grooves distinct. Clypeus 0.20 high. Chelicerae with five promarginal and five retromarginal teeth. Sternum yellowish brown, as long as wide, shield-like, with microsetae. Ocular region strongly curved, extending above clypeus, eyes: AER recurved, PER procurved, slightly wider than AER. Eye sizes and interdistances: AME 0.03, ALE 0.06, PME 0.04, PLE 0.05, AME–AME 0.02, PME–PME 0.07, AME–ALE, 0.02, PME–PLE 0.03, AME–PME 0.06, ALE–ALE 0.19, PLE–PLE 0.20, ALE–PLE contiguous. Length of legs: I 1.97 (0.59, 0.21, 0.48, 0.36, 0.33), II 1.82 (0.56, 0.19, 0.43, 0.35, 0.29), III 1.68 (0.58, 0.15, 0.45, 0.28, 0.22), IV 2.00 (0.58, 0.19, 0.48, 0.38, 0.39). TmI 0.63 and TmIV absent. Tibial spine formula: 1-1-1-1. Opisthosoma 0.97 long, 0.71 wide, oval, greenish.

***Palp*** (Fig. 3A–D). Femur unmodified, almost as long as both patella and tibia. Patella as long as tibia. Tibia widest at distal end, with one retrolateral and one dorsal trichobothria, with three apophyses; dorsal tibial apophysis almost as long as cymbium, slightly curved, tip with teeth; prolateral tibial apophysis (PTA) small, somewhat triangular in dorsal view; retrolateral tibial apophysis sclerotized, tip with serrated margin; paracymbium J-shaped, basally with setae, distal arm tip with blunt end; tegulum almost round, ventrally bulging above the subtegulum, protegulum distinct, membranous; suprategulum small, with small median suprategular apophysis, distal suprategular apophysis membranous; embolic division simple, consisting of a radix longer than wide; tailpiece slightly curved with blunt tip; median membrane short, present in between embolus and distal suprategular apophysis; embolus curved, with bifurcated tip.

**Female** (paratype SWUC-T-LIN-27-02, Fig. 5D, E): total length 1.64; carapace 0.76 long, 0.51 wide, cephalic region slightly elevated, brown, fovea, cervical and radial grooves distinct. Clypeus 0.11 high. Chelicerae with six promarginal and five retromarginal teeth. Sternum shield-shaped, as wide as long, greenish brown, with microsetae. Eyes: AER recurved, PER straight, slightly wider than AER. Eye sizes and interdistances: AME 0.04, ALE 0.06, PME 0.04, PLE 0.05, AME–AME 0.01, PME–PME 0.04, AME–ALE, 0.01, PME–PLE 0.02, AME–PME 0.05, ALE–ALE 0.21, PLE–PLE 0.23, ALE–PLE contiguous. Length of legs: I 1.73 (0.40.53, 0.20, 0.42,0.30, 0.28), II 1.59 (0.50, 0.19, 0.35, 0.29, 0.26), III 1.32 (0.39, 0.16,0.28, 0.27, 0.22), IV 1.74 (0.53, 0.16, 0.42,0.34, 0.29). TmI 0.64 and TmIV absent. Tibial spine formula: 1-1-1-1. Opisthosoma 0.96 long, 0.64 wide, oval, greenish.

***Epigyne*** (Fig. 4A–E). Epigynal plate twice as wide as long, anteriorly with round patch; ventral plate tapering ventrally; dorsal plate almost rectangular, with small groove at the center; copulatory opening located mid-ventrally at the junction of dorsal and ventral plates; copulatory ducts large, curving into one loop; spermathecae large and kidney-shaped, located anterior to the epigynal transverse slit, spaced by two diameters; fertilization ducts large and sinuous.

##### Distribution.

Known only from the type locality (Fig. 12).

#### ﻿ Tapinocyba triangularis sp. nov.

Taxon classificationAnimaliaAraneaeLinyphiidae

7F59FBB0-D080-581E-8F32-47990AB213A4

https://zoobank.org/423E1D35-63A9-4C2A-ACDC-B5675DE3F080

Figs 6 , 7 , 8 , 12

##### Type material.

***Holotype***: China • ♂; Chongqing Municipality, Jiangjin District, Tanghe Town, Gunziping Jianshanzi, Dayuandong, 28°55′46.81″N, 106°5′13.45″E, 752 m a.s.l., 2023.III.25, Wang L.Y. et al. leg.; SWUC-T-LIN-28-01. ***Paratypes***: 6♂6♀; same location data as holotype; SWUC-T-LIN-28-02 to SWUC-T-LIN-28-13 • 5♂4♀; Tanghe Town, Gunziping, Jianshanzi, Dayuandong, 28°55′46.81″N, 106°5′13.45″E, 752 m a.s.l., 2023.4.15 and 2023.IV.30., Wang L.Y. et al. leg.; SWUC-T-LIN-28-14 to SWUC-T-LIN-28-22 • 1♂; Simian Mountain, Tudi Yan Guard Management Station, 28°37′24.45″N, 106°24′6.69″E, 1126 m a.s.l., 2023.IV.27, Zhang Z.G et al. leg.; SWUC-T-LIN-28-23.

##### Etymology.

The specific epithet is derived from the Latin adjective *triangularis*, meaning “triangular” and referring to the somewhat triangular ventral part of the dorsal tibial apophysis of the male palp in retrolateral view.

##### Diagnosis.

**Figure 6.**
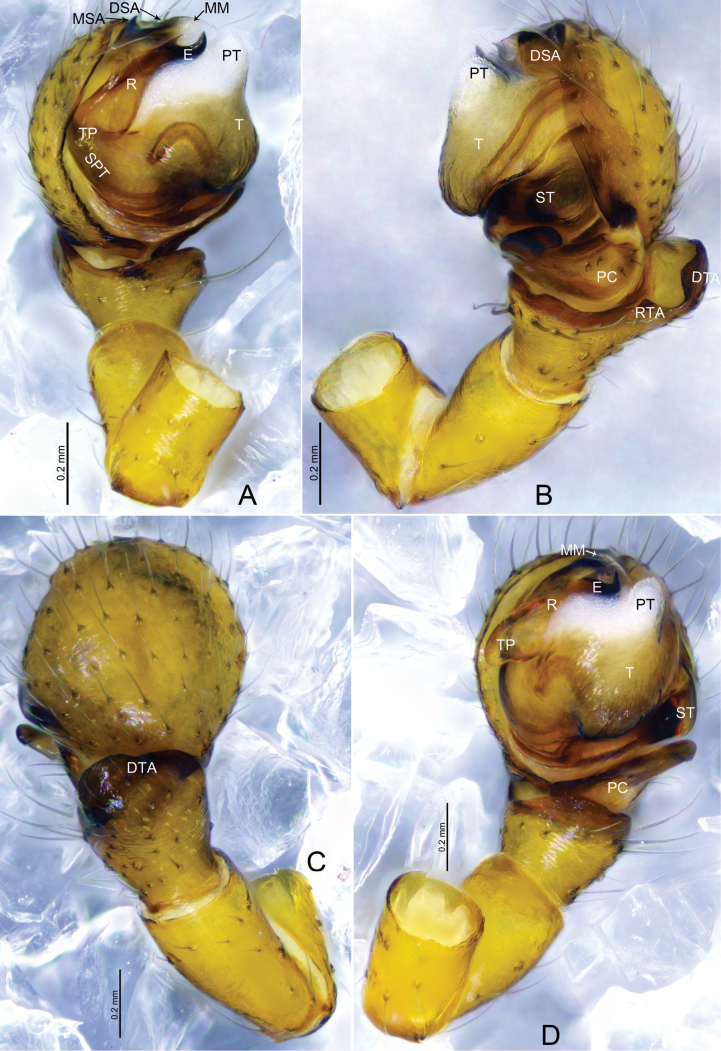
*Tapinocybatriangularis* sp. nov., male holotype **A** palp, prolateral view **B** palp, retrolateral view **C** palp, dorsal view **D** palp, ventral view. Abbreviations: DSA = distal suprategular apophysis; DTA = dorsal tibial apophysis; E = embolus; MM = median membrane; MSA = marginal suprategular apophysis; PC = paracymbium; PT = protegulum; R = radix; RTA = retrolateral tibial apophysis; SPT = suprategulum; ST = subtegulum; T = tegulum; TP = tailpiece.

**Figure 7.**
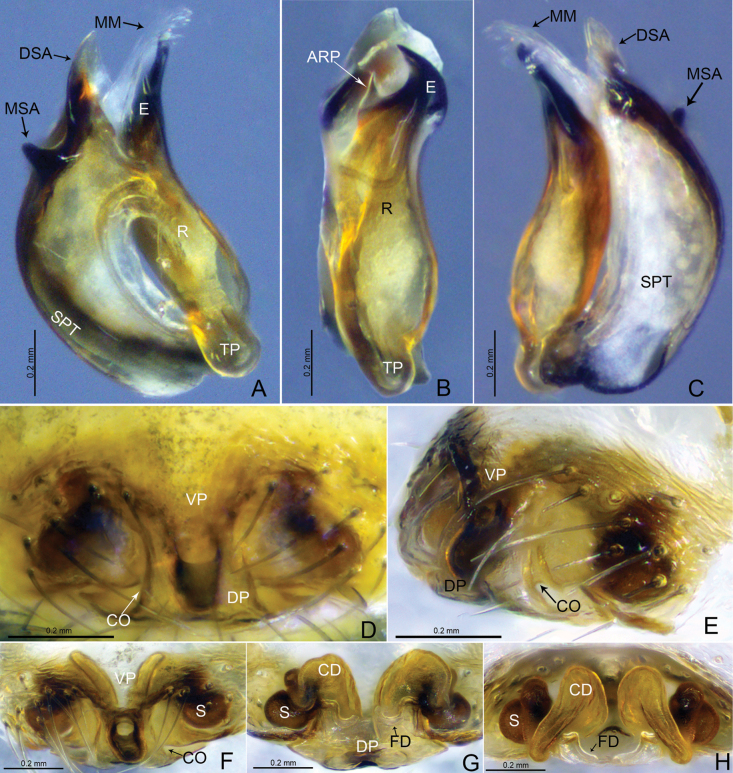
*Tapinocybatriangularis* sp. nov., male paratype (**A–C**), female paratype (**D–H**) **A–C** embolic division **D, F** epigyne, ventral view **E** epigyne, lateral view **G** vulva, dorsal view **H** vulva, anterior view. Abbreviations: ARP = anterior radical process; CD = copulatory duct; CO = copulatory opening; DP = dorsal plate; DSA = distal suprategular apophysis; E = embolus; FD = fertilization duct; MM = median membrane; MSA = marginal suprategular apophysis; R = radix; S = spermathecae; SPT = suprategulum; TP = tailpiece; VP = ventral plate.

**Figure 8.**
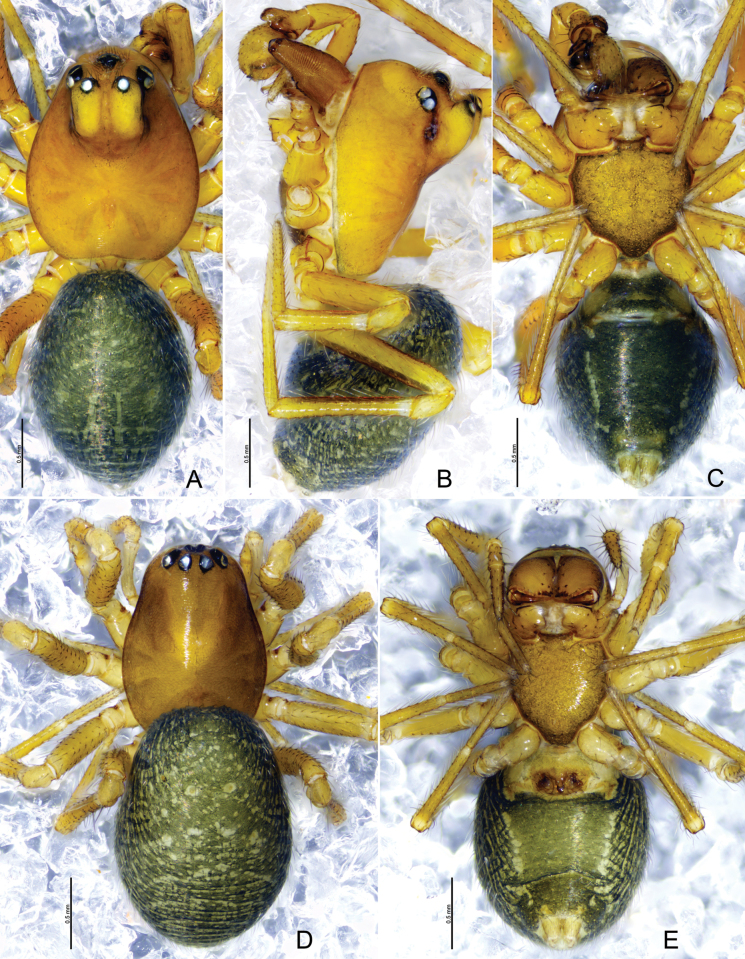
*Tapinocybatriangularis* sp. nov., male holotype (**A–C**) and female paratype (**D, E**) **A, D** habitus, dorsal view **B** habitus, lateral view **C, E** habitus, ventral view.

The male of *Tapinocybatriangularis* sp. nov. resembles *T.emei* Tanasevitch, 2018 in having the similar cephalic lobe, tibial apophyses, distal suprategular apophysis (Figs 6A–D, 7A–C; Tanasevitch 2018: figs 1–5) and resembles *T.algirica* Bosmans, 2007 in having the similar anterior radical process and embolus in male palp (Bosmans 2007: figs 153–156), but it can be distinguished by the horn-shaped embolus in *T.triangularis* sp. nov. (Fig. 8A; vs claw-shaped in *T.emei*), anterior radical apophysis present in *T.triangularis* sp. nov. (Figs 6B, 7B; vs absent in *T.emei*), and tibial apophyses present in *T.triangularis* sp. nov. (Fig. 8A; vs absent in *T.algirica*). The female of *T.triangularis* sp. nov. resembles *T.virga* sp. nov. in having the similar ventral and dorsal plate (Figs 7D–H, 10D–H) and can be distinguished by the spermathecae globular in *T.triangularis* sp. nov. (Fig. 7G, H; vs oval); copulatory opening as long as wide in *T.triangularis* sp. nov. (Fig. 7D vs longer than wide).

##### Description.

**Male** (holotype, Fig. 8A–C): total length 1.98; carapace 0.94 long, 0.75 wide, brown, cephalic lobe 0.35 long, 0.15 wide, strongly elevated, cephalic pits present at the base of cephalic lobe, fovea, cervical and radial grooves distinct. Clypeus 0.29 high. Chelicerae with five promarginal and five retromarginal teeth. Sternum yellowish brown as long as wide, shield-like, with microsetae. Eyes: AER recurved, PER procurved, slightly wider than AER, PMEs present on cephalic lobe. Eye sizes and interdistances: AME 0.05, ALE 0.07, PME 0.05, PLE 0.06, AME–AME 0.01, PME–PME 0.11, AME–ALE, 0.07, PME–PLE 0.15, AME–PME 0.13, ALE–ALE 0.37, PLE–PLE 0.39, ALE–PLE 0.01. Length of legs: I 2.49 (0.72, 0.25, 0.61, 0.50, 0.41), II 2.23 (0.66, 0.22, 0.54, 0.45, 0.36), III 1.9 (0.56, 0.22, 0.41, 0.40, 0.31), IV 2.51 (0.72, 0.23, 0.62, 0.55, 0.39). TmI 0.43 and TmIV absent. Tibial spine formula: 1-1-1-1. Opisthosoma 0.98 long, 0.72 wide, oval, greenish.

***Palp*** (holotype, Fig. 6A–D, a paratype SWUC-T-LIN-28-02, Fig. 7A–C). Femur unmodified, almost as long as both patella and tibia. Patella longer than tibia. Tibia widest at distal end, with one retrolateral and one dorsal trichobothria, with two apophyses; dorsal tibial apophysis broad, tip curved ventrally pointing towards paracymbium in retrolateral view; retrolateral tibial apophysis small, somewhat triangular; paracymbium J-shaped, basally with setae, distal arm tip with blunt end; tegulum almost round, ventrally bulging above the subtegulum, protegulum distinct, membranous; suprategulum small, with distinct median suprategular apophysis, distal suprategular apophysis membranous; embolic division simple, consisting of a radix longer than wide; tailpiece tapering with blunt tip; anterior radical process half the length of embolus, sharp with pointed end; median membrane short, present in between embolus and distal suprategular apophysis; embolus slightly curved, with pointed tip.

**Female** (paratype SWUC-T-LIN-28-03, Fig. 8D, E): total length 1.76; carapace 0.84 long, 0.60 wide, cephalic region slightly elevated, brown, fovea, cervical and radial grooves distinct. Clypeus 0.10 high. Chelicerae with six promarginal and five retromarginal teeth. Sternum shield-shaped, longer than wide, yellowish brown, with microsetae. Eyes: AER recurved, PER slightly procurved, slightly wider than AER. Eye sizes and interdistances: AME 0.03, ALE 0.06, PME 0.06, PLE 0.06, AME–AME 0.02, PME–PME 0.04, AME–ALE, 0.03, PME–PLE 0.04, AME–PME 0.04, ALE–ALE 0.26, PLE–PLE 0.29, ALE–PLE contiguous. Length of legs: I 2.1 (0.57,0.22, 0.51, 0.43, 0.37), II 1.94 (0.56, 0.18,0.47,0.39, 0.34), III 1.68 (0.51,0.21, 0.32,0.36,0.28), IV 2.21 (0.64,0.20, 0.57,0.44, 0.36). TmI 0.46 and TmIV absent. Tibial spine formula: 1-1-1-1. Opisthosoma 1.10 long, 0.75 wide, oval, greenish, dorsally with a pair of sigillae.

***Epigyne*** (Fig. 7D–H). Epigynal plate twice as wide as long; ventral plate tapering ventrally, posteriorly with round hole followed by sclerotized ventral pit on it frontal face; dorsal plate almost rectangular, longer than wide; copulatory opening located posteriorly at the base of dorsal plate; copulatory ducts large, curving into two loops; spermathecae located dorsolaterally, spaced by three diameters; fertilization ducts large and sinuous.

##### Distribution.

Known only from the type locality (Fig. 12).

#### ﻿ Tapinocyba virga sp. nov.

Taxon classificationAnimaliaAraneaeLinyphiidae

9D6B30B0-9F68-5F8E-8DE6-7BAE2D22D869

https://zoobank.org/6C72739C-2035-4788-B65B-1E72FCB0520A

**Figure 9.**
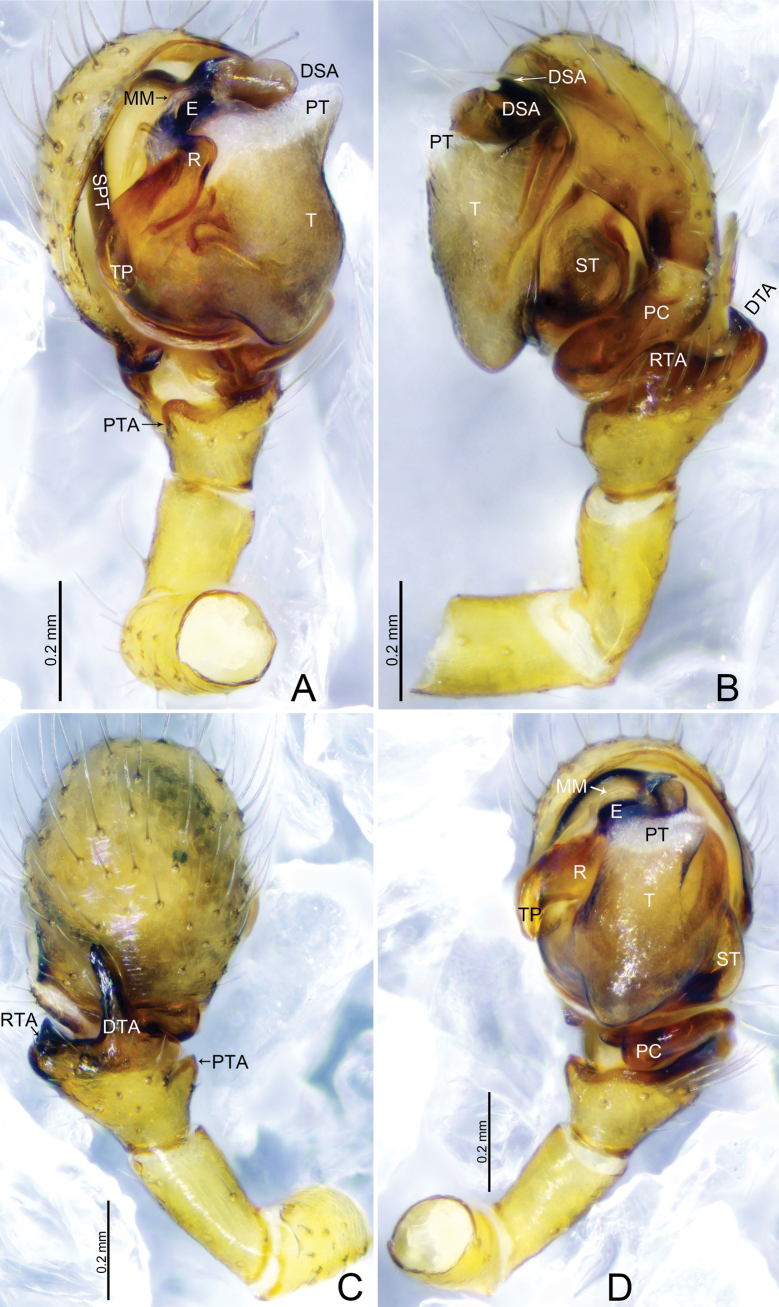
*Tapinocybavirga* sp. nov., male holotype **A** palp, prolateral view **B** palp, retrolateral view **C** palp, dorsal view **D** palp, ventral view. Abbreviations: DSA = distal suprategular apophysis; DTA = dorsal tibial apophysis; E = embolus; MM = median membrane; MSA = marginal suprategular apophysis; PC = paracymbium; PT = protegulum; PTA = prolateral tibial apophysis; R = radix; RTA = retrolateral tibial apophysis; SPT = suprategulum; ST = subtegulum; T = tegulum; TP = tailpiece.

**Figure 10.**
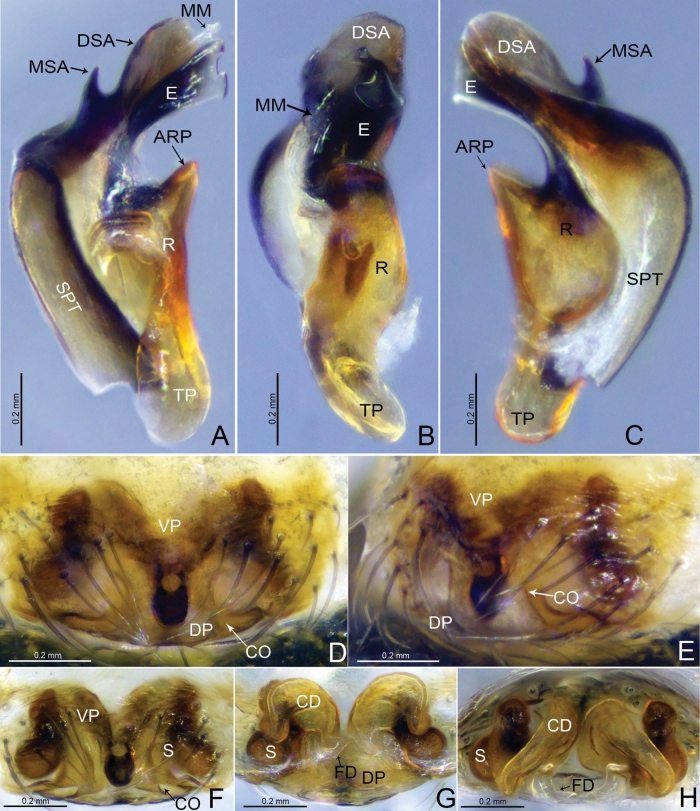
*Tapinocybavirga* sp. nov., male paratype (**A–C**), female paratype (**D–H**) **A–C** embolic division **D, F** epigyne, ventral view **E** epigyne, lateral view **G** vulva, dorsal view **H** vulva, anterior view. Abbreviations: ARP = anterior radical process; CD = copulatory duct; CO = copulatory opening; DP = dorsal plate; DSA = distal suprategular apophysis; E = embolus; FD = fertilization duct; MM = median membrane; MSA = marginal suprategular apophysis; R = radix; S = spermathecae; SPT = suprategulum; TP = tailpiece; VP = ventral plate.

**Figure 11.**
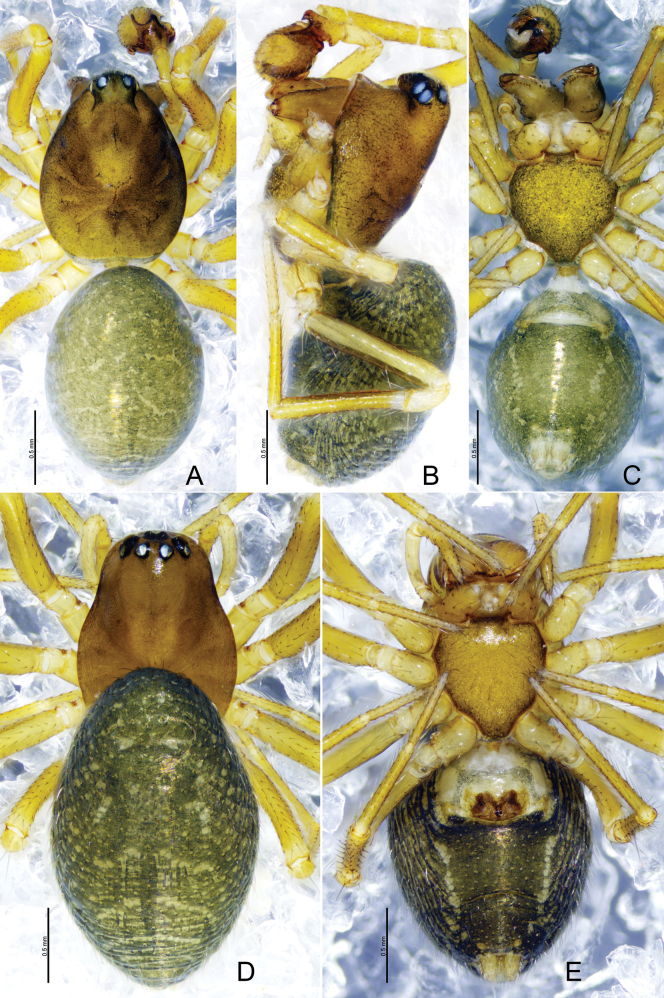
*Tapinocybavirga* sp. nov., male holotype (**A–C**) and female paratype (**D, E**) **A**, **D** habitus, dorsal view **B** habitus, lateral view **C, E** habitus, ventral view.

**Figure 12.**
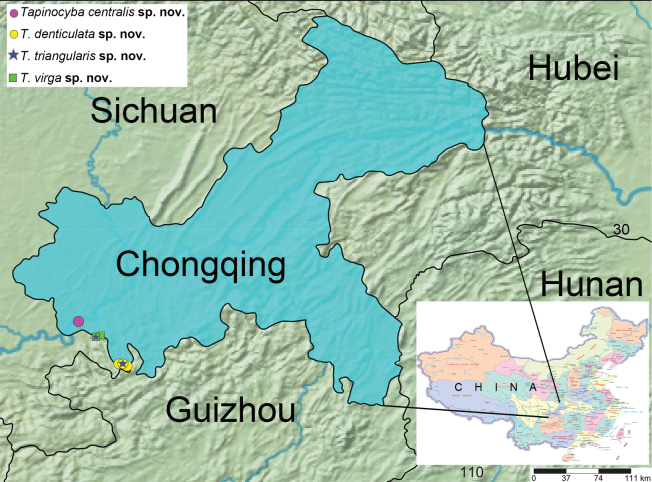
Distribution of *Tapinocyba* species in Jiangjin, Chongqing.

Figs 9 , 10 , 11 , 12

##### Type material.

***Holotype***: China • ♂; Chongqing Municipality, Jiangjin District, Tanghe Town Gunziping Jianshanzi, Dayuandong, 28°55′46.81″N, 106°5′13.45″E, 752 m a.s.l., 2023.III.5, Wang L.Y. et al. leg.; SWUC-T-LIN-29-01. ***Paratypes***: 1♂3♀; same location data as holotype; SWUC-T-LIN-29-02 to SWUC-T-LIN-29-05 • 1♂; Tanghe Town Gunziping Jianshanzi, Dayuandong, 28°55′46.81″N, 106°5′13.45″E, 752 m a.s.l., 2023.III.25, Wang L.Y. et al. leg.; SWUC-T-LIN-29-06 • 2♂; Baisha Town, Zhang Gongshan, Xiannu Cave, 28°57′10.27″N, 106°8′57.34″E, 809 m a.s.l., 2023.III.5, Wang L.Y. et al. leg.; SWUC-T-LIN-29-07 & SWUC-T-LIN-29-08 • 2♂; Tanghe Town Gunziping Jianshanzi, Dayuandong, 28°55′46.81″N, 106°5′13.45″E, 752 m a.s.l., 2023.II.5, Wang L.Y. et al. leg.; SWUC-T-LIN-29-09 & SWUC-T-LIN-29-10 • 1♀; Tanghe Town, Longyuemen, Dayuandong, Gunziping Management Station, 28°55′43.93″N, 106°5′20.81″E, 773 m a.s.l., 2023.II.5, Wang L.Y. et al. leg.; SWUC-T-LIN-29-11.

##### Etymology.

The specific epithet is derived from the Latin noun *virga*, meaning “rod” and referring to the somewhat rod-like dorsal tibial apophysis of the male palp.

##### Diagnosis.

The male of *Tapinocybavirga* sp. nov. resembles *T.praecox* (O. Pickard-Cambridge, 1873) in having the similar embolic division (Figs 9A–D, 10A–C; Hormiga 2000: fig. 27A–E, pl. 62A–F) and can be distinguished by the ocular region modified in *T.virga* sp. nov. (Fig. 11B; vs not modified); tibia with three apophyses (DTA, PTA and RTA) in *T.virga* sp. nov. (Fig. 9A, B, D; vs tibia with one apophysis); anterior radical apophysis present in *T.virga* sp. nov. (Figs 10A, C; vs absent); and embolus as long as radix with bifurcated tip in *T.virga* sp. nov. (Figs 9A, 10A; vs much shorter than radix with pointed tip). The female of *T.virga* sp. nov. resembles *T.triangularis* sp. nov. in having the similar ventral and dorsal plate (Fig. 10D–H; Fig. 7D–H) and can be distinguished by the spermathecae oval in *T.virga* sp. nov. (Fig. 10G, H; vs globular); copulatory opening longer than wide in *T.virga* sp. nov. (Fig. 10D vs as long as wide).

##### Description.

**Male** (holotype, Fig. 11A–C): total length 1.69; carapace 0.79 long, 0.59 wide, brown, cephalic region slightly elevated, cephalic pits absent, fovea, cervical and radial grooves distinct. Clypeus 0.20 high. Chelicerae with six promarginal and five retromarginal teeth. Sternum yellowish brown longer than wide, shield-like with microsetae. Ocular region strongly curved, extending above clypeus, eyes: AER recurved, PER procurved, slightly wider than AER. Eye sizes and interdistances: AME 0.03, ALE 0.04, PME 0.06, PLE 0.05, AME–AME 0.03, PME–PME 0.07, AME–ALE, 0.01, PME–PLE 0.02, AME–PME 0.07, ALE–ALE 0.19, PLE–PLE 0.21, ALE–PLE contiguous. Length of legs: I 1.88 (0.55, 0.21,0.46, 0.39,0.27), II 1.73 (0.52, 0.19, 0.41,0.33, 0.28), III 1.44 (0.41, 0.17, 0.31, 0.30, 0.25), IV 2.51 (0.56, 0.20, 0.48, 0.36,0.29). TmI 0.54 and TmIV absent. Tibial spine formula: 1-1-1-1. Opisthosoma 0.96 long, 0.66 wide, oval, greenish.

***Palp*** (holotype, Fig. 9A–D, a paratype SWUC-T-LIN-29-02, Fig. 10A–C). Femur unmodified, almost as long as both patella and tibia. Patella longer than tibia. Tibia widest at distal end, with one retrolateral and one dorsal trichobothria, with two apophyses; dorsal tibial apophysis rod-like, two-third length of tibia; prolateral tibial apophysis (PTA) thumb-shaped with tapering tip in dorsal view; retrolateral tibial apophysis D-shaped; paracymbium J-shaped, basally with setae, distal arm tip hook-shaped with blunt end; tegulum almost round, ventrally bulging above the subtegulum, protegulum distinct, membranous; suprategulum small, with distinct median suprategular apophysis, distal suprategular apophysis membranous; embolic division simple, consisting of a radix longer than wide; tailpiece slightly curved with blunt tip; anterior radical process one-third the length of embolus, with blunt tip; median membrane short, present in between embolus and distal suprategular apophysis; embolus almost as long as radix, with bifurcated tip.

**Female** (paratype SWUC-T-LIN-29-03, Fig. 11D, E): total length 1.86; carapace 0.91 long, 0.62 wide, cephalic region slightly elevated, brown, fovea, cervical and radial grooves distinct. Clypeus 0.10 high. Chelicerae with six promarginal and five retromarginal teeth. Sternum shield-shaped, longer than wide, yellowish brown with microsetae. Eyes: AER recurved, PER slightly procurved, slightly wider than AER. Eye sizes and interdistances: AME 0.03, ALE 0.06, PME 0.06, PLE 0.05, AME–AME 0.03, PME–PME 0.06, AME–ALE, 0.02, PME–PLE 0.02, AME–PME 0.05, ALE–ALE 0.26, PLE–PLE 0.28, ALE–PLE 0.01. Length of legs: I 2.1 (0.57,0.22, 0.51, 0.43, 0.37), II 1.99 (0.60, 0.23, 0.45, 0.38, 0.33), III 1.67 (0.52, 0.18, 0.36,0.35, 0.26), IV 2.22 (0.66, 0.19, 0.55, 0.46, 0.33). TmI 0.50 and TmIV absent. Tibial spine formula: 1-1-1-1. Opisthosoma 1.10 long, 0.75 wide, oval, greenish, dorsally with a pair of sigillae, ventral side greenish black.

***Epigyne*** (Fig. 10D–H). Epigynal plate two times wider than long; ventral plate tapering ventrally, posteriorly with round hole followed by sclerotized ventral pit on its frontal face; dorsal plate almost rectangular, longer than wide; copulatory opening located posteriorly at the base of dorsal plate; copulatory ducts large, curving into two loops; spermathecae located dorsolaterally, spaced by three diameters; fertilization ducts large and sinuous.

##### Distribution.

Known only from the type locality (Fig. 12).

##### Habitat.

The specimens of the species described here were collected from leaf litter in broad-leaved and coniferous forests.

## ﻿Remarks

After examining and comparing the new species with the type species of the genus *Tapinocyba* and other related species, we conclude that they share both somatic and genital similarities. The male palps exhibit comparable embolic divisions, featuring a small embolus and an anterior radical process, as described by Millidge (1979). The epigynes also display almost identical structures and shapes, except for the epigynal plate with a pit ventrally on its frontal face in *T.triangularis* sp. nov. and *T.virga* sp. nov. that is absent in all other known *Tapinocyba* species. Based on these structural and morphological affinities, we propose that all the new species presented here belong to the genus *Tapinocyba*.

## Supplementary Material

XML Treatment for Tapinocyba

XML Treatment for Tapinocyba centralis

XML Treatment for Tapinocyba denticulata

XML Treatment for Tapinocyba triangularis

XML Treatment for Tapinocyba virga

## ﻿Additional information

### Author ORCIDs

Muhammad Irfan https://orcid.org/0000-0003-0445-9612

Dai Yun https://orcid.org/0009-0005-2709-5788

Wang Lu-Yu https://orcid.org/0000-0002-5250-3473

Zhang Zhi-Sheng https://orcid.org/0000-0002-9304-1789

### Data availability

All of the data that support the findings of this study are available in the main text.
